# Supplementary material for: Consumption of Tea, Alcohol, and Fruits and Risk of Kidney Stones: A Prospective Cohort Study in 0.5 Million Chinese Adults
Source: Nutrients. 2021 Mar 29;13(4):1119. doi: 10.3390/nu13041119 (PMC8065818; doi:10.3390/nu13041119)
Supplement: Supplementary file 1 [file nutrients-13-01119-s001.pdf]

Supplemental material

Consumption of Tea, Alcohol, and Fruits and Risk of Kidney Stones: A Prospective Cohort Study in 0.5 Million Chinese Adults

Appendix Members of the China Kadoorie Biobank collaborative group 2
Table S1 Subgroup analyses of the association between tea consumption and risk of kidney stones (n = 502,621) 3
Table S2 Subgroup analyses of the association between alcohol consumption and risk of kidney stones (n = 502,621) 5
Table S3 Subgroup analyses of the association between fruit consumption and risk of kidney stones (n = 502,621) 7

Appendix Members of the China Kadoorie Biobank collaborative group

International Steering Committee: Junshi Chen, Zhengming Chen (PI), Robert Clarke, Rory Collins, Yu Guo, Liming Li (PI), Jun Lv, Richard Peto, Robin Walters. **International Co-ordinating Centre, Oxford:** Daniel Avery, Ruth Boxall, Derrick Bennett, Yumei Chang, Yiping Chen, Zhengming Chen, Robert Clarke, Huaidong Du, Simon Gilbert, Alex Hacker, Mike Hill, Michael Holmes, Andri Iona, Christiana Kartsonaki, Rene Kerosi, Ling Kong, Om Kurmi, Garry Lancaster, Sarah Lewington, Kuang Lin, John McDonnell, Iona Millwood, Qunhua Nie, Jayakrishnan Radhakrishnan, Paul Ryder, Sam Sansome, Dan Schmidt, Paul Sherliker, Rajani Sohoni, Becky Stevens, Iain Turnbull, Robin Walters, Jenny Wang, Lin Wang, Neil Wright, Ling Yang, Xiaoming Yang. **National Co-ordinating Centre, Beijing:** Zheng Bian, Yu Guo, Xiao Han, Can Hou, Jun Lv, Pei Pei, Chao Liu, Canqing Yu. **10 Regional Co-ordinating Centres: Qingdao CDC:** Zengchang Pang, Ruqin Gao, Shanpeng Li, Shaojie Wang, Yongmei Liu, Ranran Du, Yajing Zang, Liang Cheng, Xiaocao Tian, Hua Zhang, Yaoming Zhai, Feng Ning, Xiaohui Sun, Feifei Li. **Licang CDC:** Silu Lv, Junzheng Wang, Wei Hou. **Heilongjiang Provincial CDC:** Mingyuan Zeng, Ge Jiang, Xue Zhou. **Nangang CDC:** Liqiu Yang, Hui He, Bo Yu, Yanjie Li, Qinai Xu,Quan Kang, Ziyan Guo. **Hainan Provincial CDC:** Dan Wang, Ximin Hu, Jinyan Chen, Yan Fu, Zhenwang Fu, Xiaohuan Wang. **Meilan CDC:** Min Weng, Zhendong Guo, Shukuan Wu,Yilei Li, Huimei Li, Zhifang Fu. **Jiangsu Provincial CDC:** Ming Wu, Yonglin Zhou, Jinyi Zhou, Ran Tao, Jie Yang, Jian Su. **Suzhou CDC:** Fang liu, Jun Zhang, Yihe Hu, Yan Lu, , Liangcai Ma, Aiyu Tang, Shuo Zhang, Jianrong Jin, Jingchao Liu. **Guangxi Provincial CDC:** Zhenzhu Tang, Naying Chen, Ying Huang. **Liuzhou CDC:** Mingqiang Li, Jinhuai Meng, Rong Pan, Qilian Jiang, Jian Lan,Yun Liu, Liuping Wei, Liyuan Zhou, Ningyu Chen Ping Wang, Fanwen Meng, Yulu Qin,, Sisi Wang. **Sichuan Provincial CDC:** Xianping Wu, Ningmei Zhang, Xiaofang Chen,Weiwei Zhou. **Pengzhou CDC:** Guojin Luo, Jianguo Li, Xiaofang Chen, Xunfu Zhong, Jiaqiu Liu, Qiang Sun. **Gansu Provincial CDC:** Pengfei Ge, Xiaolan Ren, Caixia Dong. **Maiji CDC:** Hui Zhang, Enke Mao, Xiaoping Wang, Tao Wang, Xi zhang. **Henan Provincial CDC:** Ding Zhang, Gang Zhou, Shixian Feng, Liang Chang, Lei Fan. **Huixian CDC:** Yulian Gao, Tianyou He, Huarong Sun, Pan He, Chen Hu, Xukui Zhang, Huifang Wu, Pan He. **Zhejiang Provincial CDC:** Min Yu, Ruying Hu, Hao Wang. Tongxiang CDC: Yijian Qian, Chunmei Wang, Kaixu Xie, Lingli Chen, Yidan Zhang, Dongxia Pan, Qijun Gu. **Hunan Provincial CDC:** Yuelong Huang, Biyun Chen, Li Yin, Huilin Liu, Zhongxi Fu, Qiaohua Xu. **Liuyang CDC:** Xin Xu, Hao Zhang, Huajun Long, Xianzhi Li, Libo Zhang, Zhe Qiu.

Table S1 Subgroup analyses of the association between tea consumption and risk of kidney stones (n = 502,621)

Subgroups	Tea consumption <3 cups/day	Tea consumption ≥3 cups/day	$p_{interaction}$
Age at baseline (years)			0.426
<50			
Cases	4543	1378	
Cases/PYs (/10000)	22.36	28.71	
HR (95% CI)	1.00	0.85 (0.79, 0.90)	
50-59			
Cases	3135	1079	
Cases/PYs (/10000)	23.54	32.28	
HR (95% CI)	1.00	0.93 (0.86, 1.00)	
≥60			
Cases	1709	563	
Cases/PYs (/10000)	17.83	22.54	
HR (95% CI)	1.00	0.90 (0.81, 0.99)	
Gender			0.158
Male			
Cases	3827	1934	
Cases/PYs (/10000)	26.31	27.09	
HR (95% CI)	1.00	0.85 (0.81, 0.90)	
Female			
Cases	5560	1086	
Cases/PYs (/10000)	19.39	31.02	
HR (95% CI) [a]	1.00	0.95 (0.88, 1.02)	
BMI (kg/m^2)			0.072
<24			
Cases	5235	1837	
Cases/PYs (/10000)	21.46	31.71	
HR (95% CI)	1.00	0.90 (0.85, 0.95)	
≥24			
Cases	4152	1183	
Cases/PYs (/10000)	22.06	24.41	

Subgroups	Tea consumption <3 cups/day	Tea consumption ≥3 cups/day	$p_{interaction}$
HR (95% CI)	1.00	0.86 (0.80, 0.93)	
Fruit consumption (days/week)			0.525
<4			
Cases	7528	2561	
Cases/PYs (/10000)	24.15	34.67	
HR (95% CI)	1.00	0.89 (0.85, 0.93)	
≥4			
Cases	1859	459	
Cases/PYs (/10000)	15.42	14.11	
HR (95% CI)	1.00	0.85 (0.76, 0.95)	

Abbreviations: HR, hazard ratio; CI, confidence interval; PYs, person years.

Adjusted covariates in the models were consistent with the model 3 in Table 2 (except for the stratified variables).

[a] Further adjusted for menopausal status (premenopausal, perimenopausal, or postmenopausal).

Table S2 Subgroup analyses of the association between alcohol consumption and risk of kidney stones (n = 502,621)

Subgroups	Alcohol consumption <30.0 grams/day	Alcohol consumption ≥30.0 grams/day	p *interaction*
Age at baseline (years)			0.033
<50			
Cases	5496	425	
Cases/PYs (/10000)	23.18	30.13	
HR (95% CI)	1.00	0.83 (0.74, 0.92)	
50-59			
Cases	3876	338	
Cases/PYs (/10000)	25.11	27.67	
HR (95% CI)	1.00	0.73 (0.64, 0.82)	
≥60			
Cases	2093	179	
Cases/PYs (/10000)	18.38	25.75	
HR (95% CI)	1.00	0.85 (0.72, 1.01)	
Gender			0.477
Male			
Cases	4862	899	
Cases/PYs (/10000)	26.35	27.83	
HR (95% CI)	1.00	0.83 (0.77, 0.90)	
Female			
Cases	6603	43	
Cases/PYs (/10000)	20.58	44.37	
HR (95% CI) [a]	1.00	0.84 (0.62, 1.13)	
BMI (kg/m^2)			0.817
<24			
Cases	6475	597	
Cases/PYs (/10000)	22.93	30.51	
HR (95% CI)	1.00	0.81 (0.74, 0.89)	
≥24			
Cases	4990	345	
Cases/PYs (/10000)	22.38	25.17	

Subgroups	Alcohol consumption <30.0 grams/day	Alcohol consumption ≥30.0 grams/day	$p_{interaction}$
HR (95% CI)	1.00	0.77 (0.69, 0.87)	
Tea consumption (cups/day)			0.660
<3			
Cases	8871	516	
Cases/PYs (/10000)	21.37	30.24	
HR (95% CI)	1.00	0.77 (0.70, 0.84)	
≥3			
Cases	2594	426	
Cases/PYs (/10000)	28.76	26.28	
HR (95% CI)	1.00	0.84 (0.75, 0.95)	
Fruit consumption (days/week)			0.062
<4			
Cases	9282	807	
Cases/PYs (/10000)	25.92	29.41	
HR (95% CI)	1.00	0.78 (0.72, 0.85)	
≥4			
Cases	2183	135	
Cases/PYs (/10000)	14.83	23.16	
HR (95% CI)	1.00	0.93 (0.77, 1.12)	

Abbreviations: HR, hazard ratio; CI, confidence interval; PYs, person years.

Adjusted covariates in the models were consistent with the model 3 in Table 2 (except for the stratified variables).

[a] Further adjusted for menopausal status (premenopausal, perimenopausal, or postmenopausal).

Table S3 Subgroup analyses of the association between fruit consumption and risk of kidney stones (n = 502,621)

Subgroups	Fruit consumption <4 days/week	Fruit consumption ≥4 days/week	$p_{interaction}$
Age at baseline (years)			0.251
<50			
Cases	4780	1141	
Cases/PYs (/10000)	27.26	15.04	
HR (95% CI)	1.00	0.87 (0.81, 0.94)	
50-59			
Cases	3447	767	
Cases/PYs (/10000)	28.23	17.24	
HR (95% CI)	1.00	0.92 (0.84, 1.00)	
≥60			
Cases	1862	410	
Cases/PYs (/10000)	21.13	12.54	
HR (95% CI)	1.00	0.84 (0.74, 0.96)	
Gender			0.290
Male			
Cases	4829	932	
Cases/PYs (/10000)	29.02	18.47	
HR (95% CI)	1.00	0.86 (0.80, 0.93)	
Female			
Cases	5260	1386	
Cases/PYs (/10000)	24.00	13.51	
HR (95% CI) [a]	1.00	0.89 (0.83, 0.96)	
BMI (kg/m^2)			0.001
<24			
Cases	6029	1043	
Cases/PYs (/10000)	26.67	13.75	
HR (95% CI)	1.00	0.83 (0.78, 0.90)	
≥24			
Cases	4060	1275	
Cases/PYs (/10000)	25.46	16.52	

Subgroups	Fruit consumption <4 days/week	Fruit consumption ≥4 days/week	*p* *interaction*
HR (95% CI)	1.00	0.91 (0.85, 0.98)	
Tea consumption (cups/day)			0.525
<3			
Cases	7528	1859	
Cases/PYs (/10000)	24.15	15.42	
HR (95% CI)	1.00	0.88 (0.83, 0.93)	
≥3			
Cases	2561	459	
Cases/PYs (/10000)	34.67	14.11	
HR (95% CI)	1.00	0.84 (0.75, 0.94)	

Abbreviations: HR, hazard ratio; CI, confidence interval; PYs, person years.

Adjusted covariates in the models were consistent with the model 3 in Table 2 (except for the stratified variables).

[a] Further adjusted for menopausal status (premenopausal, perimenopausal, or postmenopausal).
